# Evaluating the Safety and Efficacy of a Non–Weight-Centric Approach to Obesity Prevention in Rural and Urban Female Adolescents: Quasi-Experimental Study

**DOI:** 10.2196/71341

**Published:** 2025-10-22

**Authors:** Areeg Zuair, Rola Jalloun, Naif Alzahrani, Fahad Alhowaymel, Esraa Merza, Bandar Alhumaidi, Mohammad Alahmadi

**Affiliations:** 1 Community Health Nursing Department, Taibah University, FGPR+CVM، Janadah Bin Umayyah Road, Medina, 42353, Saudi Arabia, 966 148618888; 2 Nutrition and Food Science Department, Taibah University, Medina, Saudi Arabia; 3 Department of Sport Science and Physical Activity, Taibah University, Medina, Saudi Arabia; 4 Department of Nursing Science, Shaqra University, Riyadh, Saudi Arabia

**Keywords:** adolescent obesity, macronutrient education, weight stigma, health literacy, Saudi Arabia, rural

## Abstract

**Background:**

Obesity is rising among Saudi adolescents, with rural females particularly underserved due to limited health education and sociocultural barriers. Global guidelines promote non–weight-centric approaches to reduce stigma. The Green Apple program delivers school-based, weight-neutral education, with an added metabolic noncommunicable disease (MNCD) prevention unit. Although previously tested in urban settings, it has not yet been evaluated among rural female students.

**Objective:**

This study aimed to examine the effectiveness of the Green Apple intervention in improving MNCD knowledge and its safety, defined as no adverse psychological (body image discrepancy and disordered eating) or behavioral (sedentary behavior) outcomes.

**Methods:**

This quasi-experimental study included 105 participants from urban and rural schools in Saudi Arabia, with 4 classes assigned to either the intervention group (Green Apple) or the enhanced intervention group (Green Apple+MNCD). Both programs were implemented in female-only classrooms by trained facilitators during regular school hours. The intervention was delivered once per week over 2 consecutive weeks (2 sessions), while the enhanced intervention included an additional third session. Linear mixed-effects models assessed intervention effects across three time points: baseline, postintervention, and 1-month follow-up.

**Results:**

The mean age across participants was 16.42 (SD 0.66) years, with a significant difference between groups: 15.97 (SD 0.41) years in the enhanced intervention group and 17.00 (SD 0.42) years in the intervention group (*P*<.001). Both interventions significantly improved knowledge across schools. The enhanced intervention (Green Apple+MNCD) group demonstrated an increase of 1.65 (95% CI 0.61-2.70; *P*<.001) from baseline to follow-up, while the intervention (Green Apple) group showed an increase of 1.26 (95% CI 0.10-2.43; *P*=.02). However, no significant between-group differences were observed at baseline (mean difference 0.20, SE 0.46; *P*=.65), postintervention (mean difference 0.79, SE 0.45; *P*=.08), or follow-up (mean difference 0.73, SE 0.49; *P*=.13). Although sedentary behavior did not significantly decrease across all schools, a significant reduction was observed in rural schools receiving the Green Apple intervention (–3.12, 95% CI –5.67 to −0.56; *P*=.02). Regarding safety outcomes, no adverse effects on body image or disordered eating were observed. A significant reduction in disordered eating symptoms was found only in urban schools receiving the enhanced intervention (–0.94, 95% CI –1.61 to –0.26; *P*=.007).

**Conclusions:**

The Green Apple program is a culturally tailored, weight-neutral intervention that improves metabolic health literacy and reduces sedentary behavior among Saudi female adolescents without harming body image or eating behaviors. By focusing on an underserved subgroup, it addresses a key gap in health promotion and aligns with global calls for stigma-free approaches. Broader regional studies are needed to assess its long-term impact.

## Introduction

As of 2020, over 100 organizations have endorsed the Joint International Consensus Statement or pledged their commitment to eliminating weight stigma [1]. These endorsements come from a diverse range of entities, including scientific societies (eg, American Society for Nutrition, UK Association for the Study of Obesity, and Obesity Australia), academic institutions, professional organizations, medical journals (eg, *Obesity*, *Obesity Reviews*, *Obesity Science and Practice*), and parliamentary groups across the globe [1]. Weight stigma has been linked to anxiety, suicidality, disordered eating, sedentary behavior, and even weight gain [2-4]. Among adolescents, weight stigma is strongly linked to disordered eating, body dissatisfaction, and psychological distress [4]. A comprehensive systematic review by Levinson et al [5] identified consistent associations between experienced, anticipated, and internalized weight stigma and harmful eating behaviors. However, most studies were cross-sectional and based on predominantly White, female samples, limiting generalizability [5]. In addition, school-based obesity prevention programs that emphasize weight loss, BMI reporting, or calorie restriction have been associated with increased body dissatisfaction and disordered eating behaviors among adolescents [5,6]. Physiologically, stigma can activate stress responses, while psychologically, it damages self-esteem and fosters body shame, leading to disordered behaviors [7,8]. These effects underscore the need for weight-neutral interventions that avoid reinforcing weight-based shame [7,8].

In response to the growing awareness of weight stigma, non–weight-centric approaches, which focus on overall health and well-being rather than weight loss, are increasingly being adopted in adolescent school-based obesity interventions [9]. These programs emphasize holistic well-being, body acceptance, intuitive eating, and joyful movement, while tracking outcomes such as blood pressure and psychological health instead of BMI [9]. Evidence suggests that non–weight-centric interventions are associated with improved metabolic health, reduced disordered eating, and greater sustainability of healthy behaviors, while also significantly decreasing internalized weight bias and body dissatisfaction [6,9].

Dugmore et al [6] conducted a meta-analysis comparing weight-neutral and traditional weight loss interventions. Weight-neutral approaches, often guided by Health at Every Size (HAES) principles, showed comparable or superior outcomes for bulimia nervosa, self-esteem, and eating behaviors, without adverse effects on physical health. They also had lower dropout rates and better adherence [6]. However, the studies were few, small, and methodologically inconsistent. Overall, the evidence supports adopting culturally sensitive, weight-neutral interventions that focus on health behaviors rather than weight loss, particularly for vulnerable adolescent populations [6].

However, balancing obesity prevention with avoiding weight-based stigma remains a global challenge, prompting calls for research beyond Western cultural contexts [1,10]. Since perceptions of obesity and weight stigma differ across languages and cultures, it is essential to explore non–weight-centric prevention strategies in non–English-speaking regions [1,10]. Increasing awareness of these cultural and linguistic differences is vital to building inclusive public health narratives that reduce stigma globally [1,10].

In Saudi Arabia, an Arabic-speaking country, adolescent obesity rates are rapidly increasing [11,12]. Recent studies report an obesity and overweight prevalence of 31.6% (2504/7931) among Saudi adolescents [13]. In response, the Saudi government has launched initiatives like the “Strategy to Combat Obesity and Promote Physical Activity in the Arab Countries” and the “RASHAKA Program,” a school-based initiative focused on increasing physical activity and reducing body weight [14,15]. Although no studies have directly measured the psychosocial consequences of these programs in Saudi Arabia, global guidance from the World Obesity Federation cautions that language used in such campaigns — such as “eliminate obesity” or “combat obesity” — can reinforce stigma by portraying individuals with higher weight as problems to be solved [1,10]. To counter this, school-based obesity prevention programs should shift focus from weight reduction to overall well-being, moving away from the “eat less, move more” narrative [10].

A recent review of obesity prevention studies in Saudi Arabia identified only 8 interventions, despite the nation’s high prevalence of adolescent obesity [16-18]. Most studies prioritized weight reduction, emphasizing obesity prevalence and associated health risks and promoting reduced portion sizes, caloric intake reduction, and increased physical activity [16,17]. Even when interventions included components aimed at fostering positive body image and reducing stigma, the primary focus remained on weight reduction and highlighting obesity prevalence and related health risks [19]. Such framing contradicts current guidance, which discourages language that may perpetuate weight stigma or promote disordered weight-control behaviors [10,20]. A 2024 cross-sectional study conducted in Saudi Arabia found that 63% of adolescents (1023/1624) were dissatisfied with their body weight [21]. The results demonstrated significant positive correlations between weight stigma, mental health issues, and BMI, with a statistically significant regional variation.

Despite increasing attention to adolescent health in Saudi Arabia, current initiatives mostly adopt a one-size-fits-all approach that overlooks the distinct needs of rural adolescents. These populations face structural and sociocultural barriers, including limited access to health education, physical activity resources, trained personnel, and specialized health care services, placing them at a disadvantage compared with urban peers [22-24]. Rapid urbanization may also be reshaping rural communities in ways that existing public health strategies have yet to address, underscoring the need for contextually adapted, weight-neutral interventions that promote health without reinforcing stigma or excluding vulnerable subgroups.

Focusing on rural female adolescents is especially important, as this subgroup encounters intersecting barriers related to geography, gender norms, and socioeconomic status [22-24]. Compared with urban girls, they experience limited mobility due to cultural expectations, as well as fewer educational and recreational opportunities, particularly in relation to structured physical activity [22-24]. These challenges are further intensified by higher poverty rates and lower levels of parental education, which can limit access to health information and preventive care [22,23]. By targeting this underserved population, this study addresses a critical gap in the literature and responds to the urgent need for culturally tailored, equitable health interventions for those most at risk of poor health outcomes. This study is the first to implement and evaluate a culturally adapted, weight-neutral school-based intervention specifically designed for and delivered to Saudi rural female adolescents.

The Green Apple program was intentionally designed as a universal school health initiative aimed at addressing the specific challenges faced by rural and urban female adolescents in Saudi Arabia. By adopting a non–weight-centric, health-focused approach, the program avoids stigmatizing language and instead promotes positive, practical health behaviors relevant to daily life. It delivers culturally sensitive, age-appropriate content in Arabic, ensuring accessibility for students in underresourced rural areas. Importantly, the program emphasizes macronutrient education, metabolic disease prevention, and strength-building exercises that do not require special facilities, making it feasible for rural schools with limited infrastructure. Accordingly, this study was conducted to evaluate the effectiveness of the Green Apple program in enhancing knowledge about MNCDs and sedentary behavior, while also monitoring safety outcomes related to body image and disordered eating symptoms.

## Methods

### Study Design and Participants

This study employed a quasi-experimental design with 4 parallel arms, incorporating blinding and nonequivalent groups. Of the total, 2 arms received the intervention (1 urban school and 1 rural school), while the other 2 arms received the enhanced intervention (1 urban school and 1 rural school). The study was conducted in 2023 at 1 urban public female high school and 1 rural public female high school in the Medina region of Saudi Arabia. Refer to Checklist 1 for the checklist used to report this study, as recommended by the TREND Group for reporting nonrandomized evaluations of behavioral and public health interventions [25].

The schools were purposefully selected by the Ministry of Education’s School Health Department to represent urban and rural populations and ensure administrative feasibility and support. The urban school was in a city center with greater access to health care services and extracurricular health programs, while the rural school served a geographically isolated population with fewer health education resources. In addition, both schools were chosen for their history of successful collaboration with the Ministry of Education, ensuring administrative support and feasibility for program implementation.

Classes within each school were assigned to one of two program versions: (1) enhanced intervention (Green Apple+MNCD): a 3-unit, non–weight-centric program that included content on the prevention of MNCDs and (2) intervention (Green Apple): a 2-unit, non–weight-centric program without MNCD content. A randomized controlled trial (RCT) design was considered during the planning phase; however, logistical, ethical, and administrative constraints necessitated the use of a quasi-experimental design instead. RCT was not feasible due to real-world constraints, including the limited number of schools and classes, especially in rural areas. For example, in the selected rural region, only 1 secondary school for female students was available, and each grade level contained only 1 class. Randomization at the individual or class level would have introduced risk of contamination and was not permissible under school policy. Therefore, a quasi-experimental design was used.

To reduce potential selection bias, first-grade students in both urban and rural schools were assigned to the enhanced intervention condition, while second-grade students were assigned to the intervention condition. This decision was guided by the understanding that second-grade students had completed a Ministry-mandated “Healthy Lifestyles” course and would likely demonstrate higher baseline knowledge. To minimize bias from this difference, the enhanced intervention was deliberately limited to first-grade students to ensure outcome comparability. In addition, a baseline assessment of knowledge revealed no significant differences between the intervention versions. To further strengthen internal validity, linear mixed-effects models were used to adjust for potential confounding factors. These models included time, school type, and intervention type as fixed effects. Although propensity score matching is a common method for reducing selection bias in quasi-experimental studies, it was not implemented due to the limited sample size and the clustered structure of the school-based data [26,27]. Instead, baseline equivalence between groups was assessed, and no significant differences were found in preintervention knowledge scores. While quasi-experimental designs limit the ability to make causal inferences compared with randomized trials, several strategies were used to enhance internal validity. These included grade-level assignment, baseline equivalence testing, and statistical covariate control. Blinding was maintained at the classroom level between intervention groups, as well as during data collection, to reduce performance and detection bias. Nonetheless, potential limitations such as residual confounding or contextual differences across classrooms cannot be fully ruled out. All participants completed self-reported measures at 3 time points: before the intervention (baseline), immediately after the program was delivered (postprogram), and 1 month after the program was delivered (follow-up). Only students who were enrolled in the participating schools, attended the classroom sessions where the intervention was delivered, and provided both informed consent (from guardians) and assent (from students) were included in the analysis. Students who did not provide consent or assent were excluded from data collection and subsequent analysis.

### Green Apple Program

Green Apple is an innovative, educational, and universal program aimed at shifting the focus from weight and obesity to the role of liver fatty acids and vascular fat in MNCDs and how lifestyle management can address conditions like diabetes and high blood cholesterol. Unlike traditional programs, Green Apple deliberately avoids terms like “obesity,” “weight reduction,” “eat less,” and “move more.” Instead, it encourages critical thinking about macronutrients and their relationship to body energy, cardiometabolic disease prevention, and lifestyle management of MNCDs.

The Green Apple program is grounded in 3 complementary theories of behavior change: the health belief model (HBM)**,** social cognitive theory (SCT)**,** and the transtheoretical model (TTM) [28-30]. These models collectively inform the program’s structure and messaging. According to HBM, adolescents are more likely to change their behaviors when they perceive themselves at risk for serious health conditions — such as diabetes, fatty liver, or stroke — and believe that actionable steps can mitigate those risks. SCT emphasizes the importance of self-efficacy and skill-building, which Green Apple fosters through experiential learning, practical lifestyle tips, and positive reinforcement. The TTM supports the idea that behavior change is a staged process, and the Green Apple program is designed to guide students from awareness to long-term action by progressively reinforcing key health concepts across sessions.

Rather than focusing on obesity or weight reduction, the program reframes the discussion around visceral fat and liver fat, highlighting their roles in MNCDs. This framing provides a more neutral, stigma-free way to promote healthy behaviors such as balanced macronutrient intake, strength-building activities, and adherence to MyPlate dietary guidelines [31,32]. The aim is to enhance students’ understanding of metabolic health while improving self-efficacy and motivation for lifelong health habits. In addition, it empowers students with knowledge and practical lifestyle tips to support family members or friends with cardiometabolic diseases without contributing to stigma for those affected by these conditions. To ensure cultural and developmental appropriateness, all materials were delivered in Arabic and tailored to the gender-specific school environment. Sessions were delivered in female-only classrooms by 2 female researchers — one with a PhD in Nursing and the other in Clinical Nutrition — both of whom have experience in teaching school-aged students. The program was delivered once per week over a 2-week period, with each session taking 1 class period (approximately 45 min). Teaching methods included active discussion, visual aids such as culturally adapted Microsoft PowerPoint slides, and non–weight-stigmatizing illustrations.

The Green Apple program was recently evaluated in a pilot study involving 69 urban students (46 boys and 23 girls; mean age 16.18, SD 0.53 y) [33]. The intervention significantly improved MNCD knowledge (*F*_1,64_=23.45; *P*<.001), with a large effect size (partial η²=0.27). Notably, no significant change in students’ body image discrepancy was observed from pre- to postintervention (*P*=.70), reinforcing the safety of this weight-neutral approach. The program also demonstrated strong feasibility, achieving a recruitment rate of 82% (95/115) and a retention rate of 72% (69/95).

Accordingly, the current program consists of 3 structured sessions (outlined in Table 1). For this pilot study, a lower-intensity version of the program was implemented to assess its effectiveness and safety.

**Table 1.** Overview of the Green Apple program curriculum. The Green Apple program is a school-based, non–weight-centric educational intervention designed to promote metabolic health literacy among adolescents. The table outlines the content structure across 3 instructional units, including core topics and the rationale for each unit. All students received units 1 and 2; however, those in the enhanced intervention also received an additional unit 3 focused on the prevention of metabolic noncommunicable diseases.

Unit	Unit title	Topics	Rationale
Unit 1 (Green Apple)	Basics of nutrition	Carbohydrates Proteins Fats	To understand the relationship between macro and micronutrients, hunger and appetite, and convenience foods and whole foods.
Unit 2 (Green Apple)	Calories and energy	Energy in the body: how is energy produced, and why do we need it? The relationship between food and energy Where is energy stored in the body, and how is it released? The relationship between physical activity and energy storage	To understand the relationship between macronutrients and energy sources, diet and exercise, and aerobic and weight-resistance exercises
Unit 3 (Enhanced Green Apple)	Metabolic chronic diseases management	Cholesterol: understanding its types and sources (beneficial and harmful), treatment with modern medicine, and lifestyle. Diabetes: understanding types of diabetes and how modern medications and lifestyle influence blood sugar control. How to help someone with MNCD^a^.	To understand the mechanisms of stroke and diabetes and possible medical and lifestyle management plans.

a MNCD: metabolic noncommunicable disease.

### Ethical Considerations

This study is part of a larger pilot initiative and was conducted in accordance with ethical standards approved by the Shaqra University Ethics Committee (approval ERC_SU_20230005). To ensure ethical treatment of participants, multiple procedures were implemented to safeguard their rights.

Before the intervention, written informed consent was obtained from the parents of all participating students. Consent was secured approximately 3 weeks before the program began and specifically covered both participation in the educational sessions and completion of the study surveys. The study’s purpose, procedures, and voluntary nature were explained to students in an age-appropriate manner through both verbal presentations made by teachers and simple written materials provided to students and parents.

Participation was entirely voluntary. Students were clearly informed that choosing not to participate or choosing to skip any survey questions would not affect their academic grades. The intervention was delivered to all students within the classroom setting as part of the school health program; however, only students who provided informed consent and assent were included in the study analyses. Confidentiality and privacy were maintained by deidentifying all data before analysis. As a gesture of appreciation, all students received a small nonmonetary token (a keychain medley) upon completing the program surveys.

### Measurements

#### Demographics

Baseline demographics included age, self-reported weight and height (if known), parents’ education level, and monthly family income. Education level was categorized for both parents as either high (college degree and above) or low (high school degree and below). However, aside from age, rural students had limited knowledge of these details and could not complete this information, so it was excluded from the analysis.

#### Physical Activity

The Arab Teens Lifestyle Study (ATLS) includes a 22-item self-reported questionnaire designed to measure weekly physical activity by reporting the types and frequency of 11 specific physical activities (eg, walking, running, weight lifting, and housework) [34]. Then, the total energy expenditure in metabolic equivalent minutes per week (METs-min/wk) is calculated for high-vigorous physical activity of 6 METs and moderate-vigorous activity of 4 METs [34]. The ATLS also contains a 10-item self-reported eating habits questionnaire designed to measure 4 positive eating habits (eg, fruit and vegetable intake) and 6 negative eating habits (eg, sugary drink and fast food consumption) [34]. Scores are calculated based on the frequency of each habit per week. Total scores for positive eating habits (0‐28) and negative eating habits (0‐35) are generated based on frequency responses (eg, the number of days per wk). The ATLS has been specifically developed for and validated among Arab adolescents, including those from Saudi Arabia [35].

#### Sedentary Behavior

The weekdays subscale of the self-reported Sedentary Behavior Questionnaire weekdays contains 9 items designed to measure sitting time during various sedentary activities (eg, watching television, playing computer or video games, listening to music, and reading books) during weekdays [36]. The average total sitting time is calculated based on 5 weekdays. Sitting ≥7 hours/day during weekdays is considered a highly sedentary behavior [37]. In our study, weekday sedentary behavior was measured at baseline and during follow-up. The Sedentary Behavior Questionnaire was originally developed for Western populations; however, its items have been culturally adapted and translated into Arabic, and it has been used among Saudi college students and adolescents with acceptable levels of reliability [33,36,38].

#### Exposure to Noncommunicable Diseases Health Education

Students’ exposure to MNCD health education topics (eg, stroke and diabetes) was measured using a 4-item self-reported (yes or no) questionnaire developed by the researchers. A total score (0‐4) was generated.

#### MNCD Knowledge

An 18-item true or false quiz was developed to assess students’ knowledge of macronutrients and their role in MNCDs (eg, liver fat, vascular fat, diabetes, and hyperlipidemia). The items were aligned with the educational objectives of the Green Apple program. To ensure content validity and cultural relevance, the quiz underwent expert review by 2 nutrition professionals and a health education specialist. Items were reviewed for accuracy, relevance, and clarity in Arabic. The quiz was also piloted with a small group of students (n=12) to confirm age-appropriateness and understanding. Revisions were made to improve wording and comprehension. The quiz was intended to measure factual knowledge across multiple distinct subtopics (eg, liver fat, sugar, insulin, and vascular fat), rather than a single unidimensional construct. A total score ranging from 0 to 18 was calculated for each participant. The need for educational intervention was then evaluated using cutoff levels recommended in the guidelines of the Food and Agriculture Organization of the United Nations [39]. Using these guidelines, an urgent need for intervention was identified if 70% or fewer participants answered correctly. A considered need was indicated when 71%‐89% of participants answered correctly. No need for intervention was noted if 90% or more participants answered correctly. This scoring method enabled a targeted assessment of the gaps in students’ knowledge of macronutrients and their role in MNCD prevention. MNCD knowledge was measured at baseline, postintervention, and during follow-up.

#### Body Image Discrepancy

Body image discrepancy was assessed using silhouettes representing 4 different body sizes [40] and a silhouette rating scale, which is a widely validated and reliable tool for assessing perceived and ideal body size among adolescents [41,42]. Silhouette scales are designed to be ethnically and culturally neutral, minimizing potential bias and ensuring applicability across diverse populations, including Saudi adolescents [43,44]. Students were asked to select the silhouette that best represented their ideal body size and the one they felt most closely resembled their current body size. The difference between the 2 selections (ideal vs current) was used to determine the degree of body image discrepancy. A negative score indicated a drive for thinness (desire for a smaller body size), while a positive score reflected a drive for fullness (desire for a larger body size) [43]. Body image discrepancy was measured at baseline, postintervention, and during follow-up.

#### Disordered Eating Symptoms

The Arabic version of the Sick-Control-One Stone-Fat-Food was used to assess disordered eating symptoms through a 5-item self-reported questionnaire with “yes or no” response options [45]. An answer of “yes”=1 and “no”=0, with a total score range from 0‐5. Disordered eating symptoms were measured both at baseline and during follow-up. The Sick-Control-One stone-Fat-Food screening tool has been translated and validated in Arabic and used successfully with adolescents [45,46].

### Statistical Approach

Descriptive statistics were first calculated for all study variables, including means and SDs for continuous variables and frequencies and percentages for categorical variables. Group differences at baseline were examined using independent samples *t* tests for continuous variables and chi-square tests for categorical variables. A series of linear mixed-effects models was used to examine the effects of time, intervention type, and school type on various outcome variables, while accounting for repeated measures across time points. The analyses were conducted using IBM SPSS Statistics (version 29) with restricted maximum likelihood, allowing for robust handling of the repeated measures design. For each model, estimated marginal means were calculated to explore pairwise comparisons between time points, intervention types, and school types. The Bonferroni correction was applied to adjust for multiple comparisons. The goodness-of-fit for each model was evaluated using the Akaike information criterion and Schwarz’s Bayesian criterion. In total, 4 outcome variables were examined: knowledge, body image discrepancy, disordered eating symptoms, and sedentary behavior. Each model included time (baseline, postintervention, or follow-up), intervention type (Green Apple-MNCD or Green Apple), and school type (urban or rural) as categorical fixed effect variables. Repeated measurements were modeled with time as the repeated factor and student ID as the subject variable to account for the correlation between repeated measurements from the same individual. An unstructured covariance matrix was used to account for variability at different time points. Pairwise comparisons were conducted for both the 2-way interaction (time×intervention type) and the 3-way interaction (time×intervention type×school type). In addition, for the knowledge model, exposure to MNCD health education was included as a continuous covariate to control baseline health literacy. However, for the body image model, knowledge was included as a continuous predictor to assess its effect on body image. For the disordered eating symptoms and sedentary behavior models, there were no additional covariates. Statistical significance was determined at *P*<.05.

### Sample Size

The sample size for this study was calculated using G*Power 3.1.9.7 software (Heinrich-Heine-Universität Düsseldorf), assuming a repeated measures ANOVA design to approximate the linear mixed model framework. A medium effect size (*f*=0.25), a significance level (α=.05), and a power (1−β=.80) were used. Although our previous pilot evaluation of the same program demonstrated a large effect size (partial *η*²=0.27, equivalent to f≈0.61), we selected a more conservative medium effect size (*f*=0.25) for the current sample size estimation [33]. This decision was based on both methodological caution and practical considerations, as we anticipated a relatively small sample due to school and class availability constraints in rural areas. The design included 2 groups and 3 time points (pre, post, and follow-up), with an assumed correlation of 0.5 among repeated measures. The analysis indicated that a total sample size of 86 participants was required to achieve sufficient power. However, when adjusted for an expected attrition rate of 20%, the required sample size increased to 108 participants.

### Missing Data

An intention-to-treat approach was applied to ensure that all participants were analyzed in their original groups, even if they had missing data at later stages. Missing data were handled using restricted maximum likelihood, which is appropriate for data assumed to be missing at random in linear mixed models [47]. This method enables the use of all available data without requiring imputation or exclusion of cases with missing values, resulting in robust parameter estimation. No imputation techniques were applied, as linear mixed models inherently accommodate incomplete data in repeated measures by using the available observations for each participant [47]. Model fit was evaluated using the Akaike information criterion, and likelihood ratio tests were performed to compare nested models. The normality of residuals and homoscedasticity was checked graphically using residual plots, and no major violations of assumptions were observed.

### Data Collection

Surveys were administered in paper-based format and completed during regular class periods under the supervision of research assistants (registered nurses), who provided clarification when necessary. Baseline surveys required approximately 45 minutes to complete, while postintervention and follow-up surveys required 15‐20 minutes. Upon collection, all surveys were collected immediately and reviewed for completeness and then entered manually into a secure electronic database (Microsoft Office Forms) by the research assistants. To minimize entry errors, data were double-checked, and any discrepancies were resolved through cross-checking.

## Results

### Sample Baseline Characteristics

#### Demographics by School Type

A total of 107 students agreed to participate in the study. Furthermore, 2 cases from the urban school were excluded due to missing identification, resulting in 105 students included in the final analysis (refer to Multimedia Appendix 1 for details on data completeness). Overall, 7% of data were missing, with the majority occurring at the follow-up stage (9%). Little’s Missing Completely at Random test indicated that the data were missing completely at random (*χ*²_111_=124.7; *P*=.18), suggesting that the missingness was unlikely to introduce systematic bias. All students contributed data at a minimum of two time points.

The mean age across all participants was 16.42 (SD 0.66) years (Table 2). A significant difference in age between the intervention groups was found (*P*<.001). Combining both urban and rural schools, the overall mean age for the enhanced intervention (Green Apple+MNCD) group was 15.97 (SD 0.41) years; for the intervention (Green Apple) group, the mean age was 17.00 (SD 0.42) years. For additional baseline characteristics by intervention type, refer to Multimedia Appendix 2.

**Table 2.** Sample sizes and mean ages of students across intervention and school types. The study includes participants from both urban and rural schools, with 2 types of interventions: enhanced intervention (Green Apple+metabolic noncommunicable disease) and intervention (Green Apple).

School type and types of intervention	Participants, n	Age (years), mean (SD)
Urban school		
Green Apple+MNCD^a^	25	15.92 (0.49)
Green Apple	15	17.13 (0.35)
Total	40	16.38 (0.74)
Rural school		
Green Apple+MNCD	34	16.10 (0.35)
Green Apple	31	16.94 (0.44)
Total	65	16.45 (0.61)
Total		
Green Apple+MNCD	59	15.97 (0.41)
Green Apple	46	17.10 (0.42)
Total	105	16.42 (0.66)

a MNCD: metabolic noncommunicable disease.

#### Physical Activity and Sedentary Behavior

Table 3 presents a comparison of urban and rural schools regarding various physical activity behaviors, exposure to MNCD health education, and the need for educational interventions. Urban students reported a higher mean sedentary time than rural students; however, this difference was not statistically significant (*P*=.09). Urban students exhibited significantly higher total energy expenditure in METs-minutes/week than rural students (*P*=.03). This was primarily due to a significantly higher energy expenditure from vigorous physical activity (*P*=.009). Although urban students reported higher energy expenditure from moderate-vigorous physical activity, this difference was not statistically significant (*P*=.17).

**Table 3.** Baseline descriptive statistics by school type (urban vs rural): physical activity, exposure to metabolic noncommunicable diseases, health education, knowledge, body image, eating disorder symptoms, and perceived need for intervention. *P* values reflect between-group comparisons using independent sample *t* tests or chi-square tests where appropriate.

Variable	Urban school	Rural school	*P* value
Physical activity behaviors, mean (SD)			
Sedentary behavior (h/d)	7.85 (3.17)	6.96 (3.68)	.09
Total-intensity physical activity (METs^a^-min/wk)	3092.62 (3025.67)	2127.11 (2217.04)	.03^b^
Vigorous-intensity physical activity (METs-min/wk)	1370.75 (2042.03)	667.11 (961.12)	.009^b^
Moderate-intensity physical activity (METs-min/wk)	1592.33 (1433.34)	1353.43 (1166.18)	.17
Exposure to MNCDs^c^ health education	3.68 (0.53)	3.38 (0.93)	.02^b^
MNCDs knowledge, mean (SD)	11.78 (2.51)	10.14 (2.34)	<.001^b^
Body image discrepancy, mean (SD)	−0.769 (0.8)	−0.23 (0.98)	.002^b^
Eating disorder symptoms, mean (SD)	1.93 (1.16)	1.37 (1.04)	.006^b^
Need of intervention assessment, n (%)			
Urgent need	21 (52.5)	57 (87.7)	<.001^b^
Considered need	19 (47.5)	8 (12.3)	—^d^
No need	0 (0)	0 (0)	—

a MET: metabolic equivalent.

b Indicates statistically significant differences (*P*<.05).

c MNCD: metabolic noncommunicable disease.

d No measurement.

#### Exposure to MNCD Health Education

Urban students reported significantly higher exposure to MNCD health education than rural participants, with a significant *P* value (*P*=.02). Furthermore, urban students reported significantly higher MNCD knowledge than rural participants (*P*<.001) (Table 3).

#### Need for Intervention Assessment

A notable disparity was observed between urban and rural schools regarding the perceived need for an intervention (Table 3). In urban schools, 53% (21/40) of participants reported an urgent need for intervention, while 48% (19/40) considered the need but did not find it urgent. In rural schools, 88% (57/65) of participants reported an urgent need, with only 12% (8/65) considering the need but not finding it urgent. This difference was significant (*P*<.001).

#### Body Image Discrepancy and Disordered Eating Symptoms

Urban students demonstrated a significantly greater drive for thinness (*P*=.002) and more disordered eating symptoms (*P*=.006) than rural students (Table 3). This suggests that urban students may be more vulnerable to body image dissatisfaction and disordered eating symptoms, highlighting a potential need for targeted interventions.

### Green Apple Intervention

#### Program Effectiveness on Knowledge Level

At all 3 time points (baseline, postintervention, and follow-up), there were no statistically significant differences between the enhanced intervention (Green Apple+MNCD) group and the original intervention (Green Apple) group in knowledge about MNCDs (all *P* values >.05; Table 4). This lack of significance may reflect the substantial overlap in core educational content between the 2 interventions, particularly regarding macronutrient understanding and behavior change strategies.

**Table 4.** Within-group school differences: changes from baseline to postintervention and to follow-up. Estimates are derived from linear mixed-effects models controlling for time and school type. Values represent adjusted mean differences with 95% CI.

Change	Change from baseline to post		Change from baseline to follow-up	
	Adjusted mean difference (95% CI)	*P* value	Adjusted mean difference (95% CI)	*P* value
On knowledge about NCDs^a^ score				
Enhanced intervention (Green Apple+MNCDs^b^)				
Urban school	2.70 (1.21 to 4.19)	<.001^c^	1.83 (0.27 to 3.39)	.02^c^
Rural school	1.00 (−0.20 to 2.20)	.13	1.48 (0.09 to 2.88)	.03^c^
All schools	1.85 (0.89 to 2.81)	<.001^c^	1.66 (0.61 to 2.70)	<.001^c^
Green Apple				
Urban school	1.86 (−0.08 to 3.81)	.06	1.22 (−0.82 to 3.25)	.44
Rural school	0.67 (−0.61 to 1.96)	.61	1.03 (−0.204 to 2.39)	.20
All schools	1.27 (0.10 to 2.43)	.02^c^	1.13 (−0.10 to 2.35)	.08
On body image discrepancy				
Enhanced Intervention (Green Apple+MNCDs)				
Urban school	0.020 (−0.59 to 0.55)	>.99	0.09 (−0.43 to 0.60)	>.99
Rural school	−0.12 (−0.59 to 0.35)	>.99	0.04 (−0.41 to 0.49)	>.99
All schools	−0.05 (−0.42 to 0.33)	>.99	0.06 (−0.29 to 0.41)	>.99
Green Apple				
Urban school	−0.01 (−0.74 to 0.72)	>.99	0.42 (−0.24 to 1.08)	.36
Rural school	−0.08 (−0.59 to 0.43)	>.99	0.19 (−0.36 to 0.56)	>.99
All schools	−0.05 (−0.49 to 0.40)	>.99	−0.26 (−0.67 to 0.14)	.35
On DE^d^ symptoms				
Enhanced intervention (Green Apple+MNCDs)				
Urban school	—^e^	—	−0.94 (−1.61 to −0.26)	.007^c^
Rural school	—	—	0.09 (−0.53 to 0.69)	.78
All schools	—	—	−0.43 (−0.88 to 0.03)	.06
Green Apple				
Urban school	—	—	0.58 (−0.30 to 1.46)	.19
Rural school	—	—	−0.07 (−0.51 to 0.65)	.80
All schools	—	—	0.25 (−0.27 to 0.78)	.34
On sedentary behavior				
Enhanced intervention (Green Apple+MNCDs)				
Urban school	—	—	−1.01 (−3.94 to 1.93)	.49
Rural school	—	—	−2.05 (−4.69 to −0.59)	.12
All schools	—	—	−1.53 (−3.50 to 0.45)	.12
Green Apple				
Urban school	—	—	−1.67 (−5.59 to 2.24)	.39
Rural school	—	—	−3.12 (−5.67 to −0.57)	.02^c^
All schools	—	—	−2.39 (−4.73 to −0.06)	.04^c^

a NCD: noncommunicable disease.

b MNCD: metabolic noncommunicable disease.

c Indicates statistically significant differences (*P*<.05).

d DE: disordered eating.

e No measurement.

Although the enhanced intervention (Green Apple*+*MNCD) group received additional disease-specific content, the intervention intensity (1 extra session) may not have been sufficient to produce a measurable advantage, especially given the short follow-up period and modest sample size (refer to Multimedia Appendix 3 for details on types of units included within each intervention).

The direction of the difference at follow-up favored the enhanced intervention (Green Apple+MNCD) group (mean difference 0.73, SE 0.49, 95% CI –0.24 to 1.71), suggesting a possible trend that may warrant further investigation in larger or longer-duration trials. The enhanced intervention (Green Apple+MNCD) led to significant improvements in knowledge about MNCDs across all schools, with an adjusted mean difference from baseline to postintervention of 1.85 (95% CI 0.89‐2.81; *P*<.001) and from baseline to follow-up of 1.66 (95% CI 0.61‐2.70; *P*<.001) (refer to Table 4 for within-group school differences in knowledge scores). In addition, when effectiveness was examined based on school type, it was found that the enhanced intervention (Green Apple+MNCD) led to significant improvements in urban schools from baseline to both postintervention with an adjusted mean difference of 2.70 (95% CI 1.21‐4.19; *P*<.001) and remained significantly higher at follow-up with an adjusted mean difference of 1.83 (95% CI 0.27‐3.39; *P*=.02). In rural schools, the enhanced intervention (Green Apple+MNCD) showed no significant improvement at postintervention with an adjusted mean difference of 1.00 (95% CI –0.20 to 2.20; *P*=.13), but a delayed significant improvement was observed at follow-up with an adjusted mean difference of 1.48 (95% CI 0.09 to 2.88; *P*=.03).

On the other hand, the intervention (Green Apple) group showed a modest overall improvement from baseline to postintervention across all schools, with an adjusted mean difference of 1.27 (95% CI 0.10‐2.43; *P*=.02). However, this was not maintained at follow-up, where the improvement was not statistically significant, with an adjusted mean difference of 1.13 (95% CI –0.10 to 2.35; *P*=.08). Furthermore, when this effectiveness was examined based on school type, it was found that in urban schools, the intervention (Green Apple) group showed marginally nonsignificant improvement at postintervention, with an adjusted mean difference of 1.86 (95% CI –0.08 to 3.81; *P*=.06), and nonsignificant improvement at follow-up, with an adjusted mean difference of 1.22 (95% CI –0.82 to 3.25; *P*=.44). Similarly, in rural schools, the intervention (Green Apple) group showed no significant improvement at postintervention (adjusted mean difference 0.67, 95% CI –0.61 to 1.96; *P*=.61) or follow-up (adjusted mean difference 1.03, 95% CI –0.204 to 2.39; *P*=.20), further supporting the conclusion of limited effectiveness.

#### Program Safety for Body Image Discrepancy and Disordered Eating Symptoms

A key aim of this study was to determine whether the program, including both the intervention (Green Apple) and the enhanced intervention (Green Apple+MNCD), maintained a neutral effect on body image discrepancy and disordered eating symptoms, as indicated by a lack of significant changes. Results across various school settings, both urban and rural, provide insight into whether these interventions unintentionally influence drive for thinness or disordered eating behaviors, which would indicate a safety concern. First, at baseline, there was no significant difference in body image discrepancy between the enhanced intervention (Green Apple+MNCD) group and the intervention (Green Apple) group (mean difference –0.05, SE 0.19, 95% CI –0.42 to 0.33; *P*=.80). This similarity at baseline confirms that the groups were comparable in this outcome before the intervention. Postintervention, the difference remained nonsignificant (mean difference –0.04, 95% CI –0.41 to 0.32; *P*=.81). However, at follow-up, a statistically significant difference was observed in favor of the enhanced intervention (Green Apple+MNCD) group (mean difference –0.37, SE 0.19, 95% CI –0.73 to –0.001; *P*=.05), indicating a greater reduction in body image discrepancy compared with the intervention (Green Apple) group.

Although the effect size was small, this finding suggests a potential delayed benefit of integrating MNCD-focused content within a weight-neutral framework. Across both intervention types and school settings, there was a general trend toward a reduction in body image discrepancy; however, none of the observed changes from baseline to follow-up reached statistical significance. For instance, in the intervention (Green Apple) group, the overall mean difference from baseline to follow-up across all schools was –0.26 (95% CI –0.67 to 0.14; *P*=.35), suggesting a modest improvement in body image perception that did not reach statistical significance. Similarly, the enhanced intervention (Green Apple+MNCD) group showed a slight increase in body image discrepancy across all schools, with a mean difference of 0.06 (95% CI –0.29 to 0.41; *P*>.99). When the data were disaggregated by school type, urban and rural schools in both interventions reflected similar nonsignificant trends. For example, in urban schools, the intervention (Green Apple) group showed a mean change of 0.42 (95% CI –0.24 to 1.08; *P*=.36), while the enhanced intervention (Green Apple+MNCD) group showed a mean change of 0.09 (95% CI –0.43 to 0.60; *P*>.99). These results suggest a generally neutral effect on body image discrepancy across both interventions, although the trends, particularly the reduction observed in the intervention (Green Apple) group, indicate potential in reducing the drive for thinness without causing psychological harm (refer to Table 5 for between-group differences and Table 4 for within-group differences in body image discrepancy).

**Table 5.** Adjusted between-group mean differences (enhanced intervention: Green Apple+MNCD vs intervention: Green Apple) at baseline, postintervention, and follow-up for key outcomes. Estimates are derived from linear mixed-effects models controlling for time and school type. Values represent adjusted mean differences with SEs and 95% CIs.

Variables	Baseline			Postintervention			Follow-up		
	Mean (SE)	95% CI	*P* value	Mean (SE)	95% CI	*P* value	Mean (SE)	95% CI	*P* value
Knowledge about MNCDs^a^	0.20 (0.46)	−0.71 to 1.12	.65	0.79 (0.45)	−0.10 to 1.7	.83	0.73 (0.49)	−0.24 to 1.71	.13
Body image discrepancy	−0.05 (0.19)	−0.42 to 0.33	.80	−0.04 (0.19)	−0.41 to 0.32	.81	−0.37 (0.19)	−0.73 to 0.001^b^	.05^b^
DE^c^ symptoms	0.30 (0.23)	−0.15 to 0.75	.18	—^d^	—	—	−0.38 (0.27)	−0.92 to 0.16	.16
Sedentary behavior	−0.07 (0.99)	−2.04 to 1.90	.94	—	—	—	0.80 (1.09)	−1.36 to 2.96	.46

a MNCDs: metabolic noncommunicable diseases.

b Indicate statistically significant differences (*P*<.05).

c DE: disordered eating.

d No measurement.

Despite the absence of statistically significant change, the observed decrease in discrepancy scores may still hold clinical relevance, as even modest reductions in the drive for thinness could reflect a shift toward healthier body image perceptions. The lack of significance may be attributed to the relatively short intervention and follow-up duration, the complexity of modifying entrenched body image attitudes, or limited statistical power to detect small effects. Second, both the enhanced intervention (Green Apple+MNCD) and the intervention (Green Apple) demonstrated no significant increases in disordered eating symptoms across various settings, supporting the safety of these weight-neutral programs (refer to Table 5 for between-group differences and Table 4 for within-group differences in disordered eating symptoms). At baseline, disordered eating symptoms were slightly higher in the enhanced intervention (Green Apple+MNCD) group than in the intervention (Green Apple) group across all schools, with a nonsignificant mean difference of 0.30 (95% CI −0.15 to 0.75; *P*=.18), suggesting comparable starting levels between the groups (Table 5). At follow-up, the enhanced intervention (Green Apple+MNCD) group showed a slight reduction in mean disordered eating symptoms, while the intervention (Green Apple) group showed a slight increase. However, neither of these changes reached statistical significance (*P*=.17 for between-group comparison at follow-up), supporting the safety of the interventions in not exacerbating disordered eating symptoms.

Across all schools, the enhanced intervention (Green Apple+MNCD) group showed a slight, nonsignificant reduction in disordered eating symptoms from baseline to follow-up (mean difference −0.43, 95% CI −0.88 to 0.03; *P*=.06). In urban schools, the enhanced intervention (Green Apple+MNCD) group demonstrated a significant reduction in disordered eating symptoms (mean difference −0.94, 95% CI −1.61 to −0.26; *P*=.007), suggesting a positive safety profile. In rural schools, the enhanced intervention (Green Apple+MNCD) group showed a nonsignificant change in symptoms from baseline to follow-up (mean difference 0.09, 95% CI −0.53 to 0.69; *P*=.78), possibly reflecting contextual differences in program delivery or participant characteristics. Similarly, the intervention (Green Apple) group in both urban and rural schools showed no statistically significant changes in symptoms, reinforcing the safety of the program’s weight-neutral design concerning disordered eating symptoms. However, it is important to note that both programs were weight-neutral; therefore, a significant difference between the 2 intervention arms was not necessarily expected. The significant reduction observed in urban schools within the enhanced intervention (Green Apple+MNCD) group may be partially explained by the higher baseline mean of disordered eating symptoms in this subgroup compared with others. This suggests that the intervention may have been particularly beneficial for students with elevated symptoms at baseline, while producing few or no significant changes in groups with lower levels of disordered eating symptoms.

#### Program Safety on Sedentary Behavior

This study aimed to assess the impact of the intervention (Green Apple) and enhanced intervention (Green Apple+MNCD) on sedentary behavior, defined as hours spent sitting per day during weekdays, with 7 or more hours defined as sedentary. A decrease in hours would suggest a positive effect on reducing sedentary behavior, supporting the interventions’ safety to not exacerbate sedentary tendencies. Overall, there was a trend toward decreased sedentary behavior across all schools in both intervention groups, with the enhanced intervention (Green Apple+MNCD) and intervention (Green Apple) groups showing reductions in mean sedentary hours from baseline to follow-up (refer to Table 5 for between-group differences and Table 4 for within-group differences in sedentary behavior). Across all schools, the enhanced intervention (Green Apple+MNCD*)* group showed a decrease in sedentary behavior from baseline (mean 10.22 h) to follow-up (mean 8.69 h), though this change was not statistically significant (mean difference −1.53, 95% CI −3.50 to 0.45; *P*=.12). In the intervention (Green Apple) group, sedentary behavior declined significantly across all schools (mean difference −2.39, 95% CI −4.73 to −0.06; *P*=.04), suggesting a positive impact.

When the data were examined by school type, both the enhanced intervention (Green Apple+MNCD) and intervention (Green Apple) groups in urban schools showed reductions in sedentary behavior from baseline to follow-up, although these changes were not statistically significant (*P*=.49 and *P*=.39, respectively). In rural schools, the intervention (Green Apple) group exhibited a statistically significant reduction in sedentary behavior (mean difference −3.12, 95% CI −5.67 to −0.57; *P*=.02). Meanwhile, the enhanced intervention (Green Apple+MNCD) group showed a nonsignificant decrease in sedentary behavior in rural schools (mean difference −1.05, 95% CI −4.69 to 2.59; *P*=.12), which supports the program’s safety. The nonsignificant difference between the 2 intervention groups regarding sedentary behavior may be attributed to the overlap in content related to calories and energy. Both interventions included the same unit discussing how energy is produced, where it is stored in the body, how it is released, and how physical activity and energy balance are related. The only distinction is that the enhanced intervention (Green Apple+MNCD) additionally linked this unit to the management of MNCDs.

## Discussion

### Principal Findings

This study found that the Green Apple program, a non–weight-centric school-based intervention, significantly improved students’ knowledge of MNCDs, reduced sedentary behavior — especially among rural students — and showed promising trends in improving body image discrepancy and disordered eating symptoms without causing harm. These findings support the effectiveness and safety of implementing culturally tailored, weight-neutral interventions in adolescent populations.

To our knowledge, this implementation trial is one of the first Arabic-language, school-based programs to evaluate a weight-neutral approach for improving understanding of MNCDs — particularly metabolic conditions like diabetes, stroke, and hyperlipidemia — while focusing on visceral fat and lifestyle changes rather than weight loss among rural and urban female adolescents. Developed using a theoretically guided process and formative research, the intervention prioritized health-promoting behaviors without reinforcing weight stigma.

### Comparison With Previous Work

The Green Apple program’s effectiveness in improving MNCD-related knowledge without exacerbating sedentary behavior tendencies can be attributed to several targeted components. First, the curriculum emphasized interactive and culturally tailored lessons that linked macronutrient intake with specific metabolic health outcomes, such as liver health and vascular fat accumulation — making abstract biomedical concepts more relevant and accessible to adolescents. Second, rather than prescribing dietary changes or promoting physical activity directly, the program employed a health literacy approach that fostered critical thinking and informed decision-making. For example, students were taught to distinguish between aerobic and resistance exercises and to identify how each affects energy metabolism. This empowered students to re-evaluate their daily habits, which may explain the observed decrease in sedentary behavior without an explicit call to increase activity. The use of relatable examples, age-appropriate language, and visual aids (eg, infographics and simplified diagrams) also played an important role in reinforcing key messages and supporting knowledge retention. The Green Apple program engaged students not only as passive recipients of information but as active participants in understanding their own health behaviors, which may have been instrumental in achieving both cognitive (knowledge) and behavioral (sedentary time) changes.

The Green Apple program was developed with a foundation in the HBM, SCT, and the TTM, aiming to enhance metabolic health literacy, self-efficacy, and staged behavior change among adolescents. Beyond these, Green Apple shares theoretical intersections with frameworks frequently used in international weight-neutral interventions. For instance, elements of self-determination theory are reflected in the program’s emphasis on autonomy (students are encouraged to make informed health decisions rather than passively follow directives), building competence (through skill-based, practical lessons), and fostering relatedness (via collaborative classroom activities) [48]. Similarly, social learning theory is embodied through its use of observational and peer-supported learning, including interactive discussions and role-modeling by program facilitators [49]. While acceptance and commitment therapy was not an explicit design framework, the program’s avoidance of weight-centric language and focus on values-driven, health-promoting behaviors may support the psychological flexibility and acceptance central to acceptance and commitment therapy–based interventions [50]. By integrating these diverse theoretical perspectives, Green Apple addresses multiple drivers of adolescent health behavior in a culturally sensitive manner.

The Green Apple program’s effectiveness in enhancing students’ understanding of MNCDs aligns with previous research highlighting the importance of health education in obesity prevention [16,51]. By focusing on the role of liver fatty acids and vascular fat in metabolic conditions rather than on weight loss, the program shifts the conversation away from potentially stigmatizing weight-centric approaches. This approach supports recent calls to reshape the global obesity narrative to recognize and reduce weight stigma [1,10,52]. The program’s success in increasing knowledge about MNCDs suggests that high school students can comprehend topics like metabolic diseases and their connection to macronutrients and body energy. This education-focused intervention demonstrates the potential of such strategies in promoting health literacy among adolescents.

Recent years have seen a global shift toward school-based health interventions that adopt weight-neutral approaches, emphasizing overall well-being, health behaviors, and body acceptance rather than weight loss or BMI reduction [53,54]. Innovative programs like Good Food for All (Australia) and Imagine HEALTH (United States) incorporate experiential activities, intuitive eating, and guided imagery to foster positive relationships with food and movement, improving psychosocial health and lifestyle behaviors without targeting weight loss [54,55]. Mindful eating curricula, such as “Eat My ABCs,” further extend these principles by teaching self-regulation and satiety awareness, leading to healthier eating behaviors in children [54]. While these programs and other international interventions share key characteristics with the weight-neutral, stigma-reducing strategies now associated with the mind-body modalities of the HAES paradigm [53,54], they are not always formally aligned with or derived from HAES.

The Green Apple program shares similarities with the HAES movement [54] in that both emphasize promoting health without focusing solely on weight, avoiding calorie counting and restrictive dieting, and encouraging a healthy relationship with food. However, there are important distinctions between the 2. The Green Apple program was specifically designed for school settings and is driven by the goal to increase metabolic health literacy among adolescents, particularly in relation to macronutrients, energy metabolism, and lifestyle-related noncommunicable diseases (eg, diabetes and hypertension). In contrast, HAES is a broader, philosophically driven approach that promotes body inclusivity, intuitive eating, and joyful movement across all populations, regardless of age, gender, or health status [53,54]. While HAES has been praised for reducing weight stigma and supporting psychological well-being, some critics argue that its generalizability to targeted disease prevention (eg, metabolic syndrome) may be limited [56,57]. On the other hand, Green Apple’s structured and curriculum-based model offers clear educational content aligned with disease prevention but may require more rigorous implementation and contextual adaptation.

Furthermore, the Green Apple program may be more culturally adaptable in non-Western contexts, such as Saudi Arabia, where public health messages around metabolic disease are often aligned with national health priorities [14,15]. In contrast, HAES’s strong focus on body autonomy and its rejection of medical weight loss may face cultural or institutional resistance in certain settings. In summary, both programs focus on health promotion beyond weight loss, but the Green Apple program — specifically, the enhanced intervention (Green Apple+MNCD) used in our study— uniquely targets metabolic health literacy and prevention, making it more tailored for specific outcomes related to metabolic disease management in adolescent females. In a previous pilot study, the Green Apple+MCND combination showed greater pre-post effectiveness on knowledge than the Green Apple program alone among male and female urban adolescents [33]. Thus, the findings from this study contribute to the literature by highlighting the effectiveness of this program among rural students and its sustainability at follow-up.

Future studies should explore the program’s effectiveness among rural male adolescents and its sustainability among urban male adolescents. Notably, rural students in this study demonstrated a delayed improvement in nutrition knowledge, with no significant gains immediately following the intervention but a marked increase at follow-up. This pattern may indicate a need for more time to absorb and consolidate new information, particularly among populations with lower baseline health literacy [22,58]. Overall, the Green Apple program demonstrated its universal acceptability among rural female adolescents and its long-term effectiveness during follow-up among both rural and urban students.

The trend reduction in sedentary behavior observed among participants is particularly noteworthy, given the high prevalence of sedentary lifestyles among Saudi youth [59]. At baseline, all schools exhibited sedentary behavior (over 7 h of sitting daily). By follow-up, sedentary time decreased by 1‐3 hours per weekday, despite the program not explicitly instructing students to reduce sitting time or increase physical activity. Several mechanisms may explain this outcome. First, the program emphasized the physiological relationship between macronutrients and energy usage, highlighting how different types of physical activity (eg, aerobic vs resistance training) impact health. This knowledge-based approach may have fostered greater internal motivation and self-efficacy to engage in movement, leading students to make conscious efforts to reduce sedentary time. Second, increased awareness of energy balance and muscle health could have encouraged students to re-evaluate their daily habits, choosing more active routines in response to improved health literacy. Similar findings have been observed in other weight-neutral interventions [54]. For example, a pilot RCT involving Latino adolescents with BMIs >95th percentile found that those who attended 12 weekly sessions of a non–weight-centric lifestyle education program called H.E.A.L.T.H. that incorporated guided imagery experienced significant reductions in leisure sedentary behavior (*P*<.05) compared with adolescents (n=14) who attended the same program with digital storytelling sessions [55]. Future studies should further explore these mechanisms by assessing not only the types and duration of physical activity adopted but also changes in students’ beliefs, confidence, and motivations regarding movement. To sum up, these findings support the potential of non–weight-centric school-based interventions to meaningfully reduce sedentary time through education and empowerment, rather than prescriptive behavioral mandates.

Finally, the promising trends in decreasing body image discrepancy and disordered eating symptoms in this study are particularly noteworthy, given the potential negative consequences of weight stigma and body dissatisfaction on adolescent mental health [2,60]. Although these changes were not statistically significant, they may still be clinically meaningful and suggest that weight-neutral approaches like the Green Apple program can support the development of positive body image and healthy eating behaviors without exacerbating weight concerns. These findings highlight the potential of such interventions to contribute to the prevention of disordered eating symptoms, even in the absence of immediate measurable effects. This aligns with recent research supporting the efficacy of weight-inclusive interventions in enhancing psychological well-being and reducing disordered eating behaviors [6,54,60]. For example, a meta-analysis found nonsignificant trends toward improvements in drive for thinness and body dissatisfaction with weight-neutral interventions [6]. However, consistent with our study, a statistically significant improvement in disordered eating symptoms was observed within one of the weight-neutral intervention groups [6].

This study’s results also highlight the importance of tailoring interventions to specific cultural contexts. The observed differences between urban and rural schools highlight the need for tailored interventions based on regional disparities. Urban students reported higher levels of knowledge and exposure to health education, along with higher body image discrepancy and disordered eating symptoms, while rural students exhibited a greater need to increase their knowledge about MNCDs. Body image is strongly shaped by social and cultural influences, and this program offers a valuable starting point for future interventions that aim to include more tailored strategies for addressing body image concerns, particularly in rural settings. This study observed an average discrepancy of less than 1 body size among students, which is consistent with previous findings suggesting that body image discrepancy tends to be low among rural adolescents [61,62]. As such, future iterations of the Green Apple program may benefit from further customization to address the specific needs of rural populations, ensuring that interventions are both accessible and effective across diverse settings. Furthermore, the use of silhouette scales, while widely accepted for body image assessment, may be limited by participants’ memory effects. Damasceno et al [63] found that participants often selected the same or very similar silhouettes within a 15-day interval, suggesting that repeated exposure to the same silhouettes over short periods can artificially inflate reliability and reduce sensitivity to actual change. This may have contributed to the lack of observed changes in body image over time.

Overall, the results in both settings demonstrate the potential for adapting weight-neutral approaches to non-Western cultures. This is particularly relevant given the limited research on obesity prevention in Saudi Arabia and the need for culturally appropriate interventions. The program’s effectiveness in both urban and rural schools further underscores its adaptability and potential for widespread implementation.

However, the observed differences between urban and rural schools warrant further exploration. Urban students generally had higher baseline levels of health literacy, greater previous exposure to health education, and better access to digital and extracurricular resources, which may have facilitated quicker knowledge gains and behavioral changes [58,64]. In contrast, rural students demonstrated a delayed improvement in nutrition knowledge, with gains becoming evident only at the follow-up stage. This delay may reflect disparities in educational resources, differences in school infrastructure, or cultural variations in how health information is received and processed [58,64]. These findings emphasize the need to tailor the program’s delivery with reinforcement strategies to better support students in underresourced rural settings and reduce health literacy gaps between regions [58,64].

### Public Health Policy Recommendations

The findings of this study suggest a significant need for policy reform in school-based health interventions, particularly in how obesity prevention is addressed in Saudi Arabia.

First, adopt non–weight-centric frameworks in school health programs. Current school health initiatives should transition from weight-focused models to non–weight-centric approaches that emphasize metabolic health. Rather than using BMI as the primary indicator of health, schools can implement annual wellness screenings that assess cardiovascular fitness, physical activity habits, and dietary quality [1,65-67]. For instance, in collaboration with schools, community health nurses, physical education staff, and local primary health care centers can adopt metrics such as heart rate recovery, physical endurance, and nutrition intake to provide a more holistic and less stigmatizing view of adolescent health.

Second, revise educational content to use stigma-free, health-promoting language. Health education policies should be revised to eliminate language that links health behaviors explicitly to weight loss. Instead, curricula should emphasize building healthy habits, such as engaging in enjoyable physical activity, eating diverse whole foods, and understanding how nutrition supports organ health [1,3]. For example, Ministry of Education health textbooks should be updated, and teacher development workshops on inclusive language should be delivered. These workshops can include scenario-based training and feedback sessions to help educators identify and eliminate implicit weight bias in their teaching [66,68].

Third, tailor health interventions to address regional disparities. As this study indicates, rural areas often lack access to tailored health education programs. To bridge this gap, mobile regional health directorates can deploy school health units to deliver interactive workshops and preventive screenings in underserved schools. In addition, rural teachers can be trained as “health ambassadors” in collaboration with local universities, ensuring consistent delivery of health messages that are culturally and contextually appropriate. Pilot programs in targeted rural regions can serve as models for broader scale-up [10,69,70].

Fourth, support long-term sustainability through policy and resource allocation. Health policies should ensure that resources are allocated for ongoing support, including teacher training and curriculum development, to maintain the positive outcomes seen in short-term interventions [10,66,68].

Fifth, encourage research and innovation in weight-neutral health promotion. Government agencies and research institutions should allocate additional resources to support studies that explore weight-neutral approaches to health promotion and obesity prevention [10,66]. National grants can prioritize school-based interventions that focus on improving metabolic indicators without relying on weight loss as an outcome. In addition, research institutions can be encouraged to collaborate with school systems to cocreate evidence-based, student-centered health programs. Finally, evaluating the Green Apple program’s adaptability to other adolescent health issues, such as iron deficiency anemia, malnutrition, or diabetes management, could broaden its impact and support more comprehensive school-based health education efforts, especially given the limited availability of evidence-based interventions targeting metabolic health in the current Saudi literature [71].

### Limitations and Recommendations

While this study presents promising results, several limitations must be acknowledged. First, the relatively short follow-up period limits our ability to assess the long-term impact and sustainability of the intervention. Some of the nonsignificant findings — particularly in outcomes such as body image discrepancy and disordered eating symptoms — may be attributed to the brief duration of the intervention, which may not have been sufficient to elicit measurable changes in complex psychosocial constructs. Future research should incorporate longer follow-up periods to better capture delayed or long-term effects. Second, the modest sample size may have reduced the statistical power to detect small but meaningful effects, especially in subgroup analyses and interaction terms. Finally, the similarity in content between the 2 intervention arms — both of which adhered to a non–weight-centric framework — may have reduced the likelihood of observing substantial between-group differences, as both emphasized promoting healthy behaviors without explicitly targeting weight loss or body image modification. In addition, since this study focused only on female students in Saudi Arabia, its findings may not be generalizable to other populations. Further research is needed to test similar interventions across diverse cultural settings and among different gender groups.

Despite these limitations, the study provides a significant contribution to adolescent obesity prevention literature, particularly in non-Western contexts. The Green Apple program’s success suggests that weight-neutral, culturally adapted interventions can effectively promote health and well-being among Saudi female adolescents. Its impact on increasing MNCD-related knowledge and its promising trends in reducing sedentary behavior, body image discrepancy, and eating disordered symptoms reinforce the value of moving away from weight-centric approaches to obesity prevention.

Overall, these findings add to the growing evidence in support of weight-inclusive health promotion strategies and underscore the need for culturally appropriate interventions in addressing global health challenges.

### Conclusions

This study demonstrated that the Green Apple program, a school-based intervention grounded in a non–weight-centric approach, effectively enhanced adolescents’ health behaviors and psychosocial outcomes. Specifically, the enhanced intervention (Green Apple+MNCD) led to significant improvements in knowledge about MNCDs across all schools. In addition, the Green Apple program without the MNCD session resulted in a notable reduction in sedentary behavior by −3.12 hours per day among rural students and a significant overall reduction of −2.39 hours per day across rural and urban schools. Both versions of the program showed promising trends in improving body image discrepancy and disordered eating symptoms without causing harm. At follow-up, a statistically significant difference was observed in favor of the enhanced intervention (Green Apple + MNCD) group, indicating a greater reduction in body image discrepancy compared with the Green Apple group. Furthermore, in urban schools, only the enhanced intervention (Green Apple+MNCD) group demonstrated a significant reduction in disordered eating symptoms. These findings support the effectiveness and safety of implementing culturally tailored, weight-neutral interventions in adolescent populations.

Future research should pursue longitudinal studies to assess the sustainability of knowledge gains and behavioral changes over extended periods. RCTs are recommended to confirm the program’s effectiveness in broader and more diverse populations, including male adolescents and students in other regions of Saudi Arabia. In addition, implementation research is needed to evaluate the program’s scalability, feasibility, and cost-effectiveness in real-world school settings. Specific priority areas include adapting the intervention to different cultural or socioeconomic contexts, integrating digital tools to enhance reach and engagement, and examining the role of school infrastructure and policy in supporting long-term behavior change. For instance, a mobile app could track daily activity and provide visual feedback on sedentary behavior, while an online platform could host culturally tailored modules on nutrition and chronic disease prevention, accessible to both students and parents. These digital enhancements would not only broaden the program’s reach but also increase engagement and retention of health messages across diverse student populations. Addressing these areas will help inform national strategies aimed at preventing metabolic chronic diseases through inclusive, sustainable, and culturally sensitive school-based interventions.

## Supplementary material

10.2196/71341 Multimedia Appendix 1 Details on data completeness.

10.2196/71341 Multimedia Appendix 2 Baseline characteristics between types of intervention.

10.2196/71341 Multimedia Appendix 3 Details on types of units included within each intervention.

10.2196/71341 Checklist 1 TREND Statement checklist.

## Abbreviations

ATLS: Arab Teens Lifestyle Study
HAES: Health at Every Size
HBM: health belief model
MET: metabolic equivalent
MNCD: metabolic noncommunicable disease
RCT: randomized controlled trial
SCT: social cognitive theory
TTM: transtheoretical model
