# Supplementary material for: Evaluating the Safety and Efficacy of a Non–Weight-Centric Approach to Obesity Prevention in Rural and Urban Female Adolescents: Quasi-Experimental Study
Source: Interact J Med Res. 2025 Oct 22;14:e71341. doi: 10.2196/71341 (PMC12543216; doi:10.2196/71341)
Supplement: Multimedia Appendix 1 [file ijmr-v14-e71341-s001.docx]

| **Data Completeness for intervention Participations** | | | | |  |
| --- | --- | --- | --- | --- | --- |
| **Variable** |  | **Baseline**  ***n*** | **Post**  ***n*** | **Follow-up**  ***n*** | **No data at all**  ***n*** |
| MCNDs Knowledge | Enhanced Intervention (Green Apple + MNCDs) | 59 | 53 | 45 | 0 |
|  | Intervention (Green Apple) | 46 | 42 | 43 | 0 |
|  |  | **105** | **95** | **88** |  |
| Disordered Eating Symptoms | Enhanced Intervention (Green Apple + MNCDs) | 58 | non | 45 | 0 |
|  | Intervention (Green Apple) | 45 | non | 43 | 0 |
|  |  | **103** |  | **88** |  |
| Body Image Discrepancy | Enhanced Intervention (Green Apple + MNCDs) | 58 | 50 | 45 | 0 |
|  | Intervention (Green Apple) | 42 | 37 | 43 | 0 |
|  |  | **100** | **87** | **88** |  |
| Sedentary Behavior | Enhanced Intervention (Green Apple + MNCDs) | 58 | non | 45 | 0 |
|  | Intervention (Green Apple) | 45 | non | 42 | 0 |
|  |  | **103** |  | **87** |  |
| All the 105 students attended the Intervention | |  |  |  |  |
| No student had completely missing data across all time points or variables | |  |  |  |  |
