# Supplementary material for: Evaluating the Safety and Efficacy of a Non–Weight-Centric Approach to Obesity Prevention in Rural and Urban Female Adolescents: Quasi-Experimental Study
Source: Interact J Med Res. 2025 Oct 22;14:e71341. doi: 10.2196/71341 (PMC12543216; doi:10.2196/71341)
Supplement: Multimedia Appendix 2 [file ijmr-v14-e71341-s002.docx]

| Baseline Characteristics Between Types of Intervention | | | | | | |
| --- | --- | --- | --- | --- | --- | --- |
| Variables | Types of Intervention | N | Mean | Std. Deviation |  | *P*- Value |
| Age | Enhanced Intervention (Green Apple + MNCDs) | 59 | 15.97 | 0.41 |  | < 0.001 |
|  | Intervention (Green Apple) | 46 | 17.10 | 0.42 |  |  |
|  |  | 105 | 16.42 | 0.66 |  |  |
| MCNDs Knowledge | Enhanced Intervention (Green Apple + MNCDs) | 59 | 11.03 | 2.06 |  | 0.21 |
|  | Intervention (Green Apple) | 46 | 10.41 | 3.00 |  |  |
| Disordered Eating Symptoms | Enhanced Intervention (Green Apple + MNCDs) | 58 | 1.72 | 1.04 |  | 0.15 |
|  | Intervention (Green Apple) | 45 | 1.40 | 1.19 |  |  |
| Body Image Discrepancy | Enhanced Intervention (Green Apple + MNCDs) | 58 | -0.52 | 0.86 |  | 0.34 |
|  | Intervention (Green Apple) | 42 | -0.33 | 1.05 |  |  |
| Sedentary Behavior | Enhanced Intervention (Green Apple + MNCDs) | 58 | 10.08 | 4.90 |  | 0.42 |
|  | Intervention (Green Apple) | 45 | 10.27 | 4.73 |  |  |
