# Supplementary material for: Evaluating the Safety and Efficacy of a Non–Weight-Centric Approach to Obesity Prevention in Rural and Urban Female Adolescents: Quasi-Experimental Study
Source: Interact J Med Res. 2025 Oct 22;14:e71341. doi: 10.2196/71341 (PMC12543216; doi:10.2196/71341)
Supplement: Multimedia Appendix 3 [file ijmr-v14-e71341-s003.docx]

| **Types of Unites Included Within Each Intervention** | | | |  |
| --- | --- | --- | --- | --- |
| **Unit** | **Unit Title** | **Topics** | **Green Apple**  **(Intervention)** | **Green Apple + MNCDs**  **(Enhanced Intervention)** |
| Unit 1 | Basics of Nutrition | 1. Carbohydrates 2. Proteins 3. Fats | Yes | Yes |
| Unit 2 | Calories and Energy | 1. Energy in the body: How is energy produced, and why do we need it? 2. The relationship between food and energy 3. Where is energy stored in the body, and how is it released? 4. The relationship between physical activity and energy storage | Yes | Yes |
| Unit 3 | Metabolic Chronic Diseases Management | 1. Cholesterol: Understanding its types and sources (beneficial and harmful), treatment with modern medicine, and lifestyle. 2. Diabetes: Understanding types of diabetes and how modern medications and lifestyle influence blood sugar control.  3. How to help someone with MNCD. | NO | Yes |
|  | | | |  |
