# Supplementary material for: Evaluating the Safety and Efficacy of a Non–Weight-Centric Approach to Obesity Prevention in Rural and Urban Female Adolescents: Quasi-Experimental Study
Source: Interact J Med Res. 2025 Oct 22;14:e71341. doi: 10.2196/71341 (PMC12543216; doi:10.2196/71341)
Supplement: Checklist 1 [file ijmr-v14-e71341-s004.docx]

| **The checklist** used to report this study, as recommended by the TREND Group for reporting nonrandomized evaluations of behavioral and public health interventions | | | | |
| --- | --- | --- | --- | --- |
| **Paper Section/Topic** | **Item No** | **Descriptor** | **Reported?** | |
|  |  |  | **√** | **section** |
| Title and Abstract | 1 | • Information on how unit were allocated to interventions | √ | Abstract |
|  |  | • Structured abstract recommended | √ | Abstract |
|  |  | • Information on target population or study sample | √ | Abstract |
| Background | 2 | • Scientific background and explanation of rationale | √ | Introduction |
|  |  | • Theories used in designing behavioral interventions | √ | Methods → Intervention |
| Participants | 3 | • Eligibility criteria for participants, including criteria at different levels in recruitment/sampling plan (e.g., cities, clinics, subjects) | √ | Methods → Study Design and Participants |
|  |  | • Method of recruitment (e.g., referral, self-selection), including the sampling method if a systematic sampling plan was implemented | √ | Methods → Study Design and Participants |
|  |  | • Recruitment setting | √ | Methods → Study Design and Participants |
|  |  | • Settings and locations where the data were collected | √ | Methods → Study Design and Participants |
| Interventions | 4 | • Details of the interventions intended for each study condition and how and when they were actually administered, specifically including: | √ | Methods → Green Apple Program |
|  |  | ○ Content: what was given? | √ | Methods → Table 1 |
|  |  | ○ Delivery method: how was the content given? | √ | Methods → Green Apple Program |
|  |  | ○ Unit of delivery: how were the subjects grouped during delivery? | √ | Methods → Green Apple Program |
|  |  | ○ Deliverer: who delivered the intervention? | √ | Methods → Green Apple Program |
|  |  | ○ Setting: where was the intervention delivered? | √ | Methods → Green Apple Program |
|  |  | ○ Exposure quantity and duration: how many sessions or episodes or events were intended to be delivered? How long were they intended to last? | √ | Methods → Green Apple Program |
|  |  | ○ Time span: how long was it intended to take to deliver the intervention to each unit? | √ | Methods → Green Apple Program |
|  |  | ○ Activities to increase compliance or adherence (e.g., incentives) | √ | Methods →Ethical Considerations |
| Objectives | 5 | • Specific objectives and hypotheses | √ | Introduction |
| Outcomes | 6 | • Clearly defined primary and secondary outcome measures | √ | Introduction |
|  |  | • Methods used to collect data and any methods used to enhance the quality of measurements | √ | Method |
| Sample Size | 7 | • How sample size was determined | √ | Methods → Sample size |
|  |  | • Explanation of interim analyses and stopping rules (if applicable) | x | NA |
| Assignment Method | 8 | • Unit of assignment (e.g., individual, group, community) | √ | Methods → Study Design and Participants |
|  |  | • Method used to assign units to study conditions (e.g., randomization, matching) | √ | Methods → Study Design and Participants |
|  |  | • Inclusion of aspects such as blocking, stratification, minimization, and restrictions |  | NA |
| Blinding (Masking) | 9 | • Whether participants, interventionists, and outcome assessors were blinded to study condition assignment | √ | Methods → Study Design and Participants |
| Unit of Analysis | 10 | • Description of the smallest unit being analyzed to assess intervention effects (e.g., individual, group, community) | √ | Methods → Study Design and Participants |
|  |  | • If the unit of analysis differed from the unit of assignment, methods used to account for this (e.g., adjusting standard error estimates) | x | NA |
| Statistical Methods | 11 | • Statistical methods used to compare study groups for primary outcome(s) | √ | Methods → Statistical Approach |
| Results |  | • Methods for additional analyses (e.g., subgroup analyses, adjusted analyses) | √ | Methods → Statistical Approach |
|  |  | • Methods for imputing missing data (e.g., multiple imputation) | √ | Methods → Missing Data |
|  |  | • Statistical software or programs used | √ | Methods → Statistical Approach |
| Participant Flow | 12 | • Flow of participants through each stage of the study: enrollment, assignment, intervention exposure, follow-up, and analysis | √ | Multimedia Appendix 2 |
|  |  | • Numbers of participants screened for eligibility, found eligible, approached for consent, and enrolled | √ | Multimedia Appendix 3 |
|  |  | • Numbers assigned to each study condition | √ | Multimedia Appendix 4 |
|  |  | • Number who received intended intervention | √ | Multimedia Appendix 5 |
|  |  | • Number of follow-ups completed | √ | Multimedia Appendix 6 |
|  |  | • Numbers analyzed | √ | Multimedia Appendix 7 |
|  |  | • Description of protocol deviations from study as planned, along with reasons | √ | Methods → Study Design and Participants |
| Recruitment | 13 | • Dates defining the periods of recruitment and follow-up | √ | Methods → Study Design and Participants |
| Baseline Data | 14 | • Baseline demographic and clinical characteristics of participants in each study condition | √ | Table 2, 3, 4, 5 |
|  |  | • Baseline characteristics relevant to specific disease prevention research | √ | Table 2, 3, 4, 5 |
|  |  | • Baseline comparisons of those lost to follow-up and retained | √ | Table 2, 3, 4, 5 |
|  |  | • Comparison between study population at baseline and target population | x | NA |
| Baseline Equivalence | 15 | • Data on study group equivalence at baseline and statistical methods used to control for baseline differences | √ | Methods → Study Design and Participants |
| Numbers Analyzed | 16 | • Number of participants (denominator) included in each analysis • Whether the analysis was by “intention to treat” or, if not, description of how non-compliers were treated in the analyses | √ | Methods → Missing Data |
|  |  | • Indication of whether the analysis strategy was “intention to treat” or, if not, description of how non-compliers were treated in the analyses | √ | Methods → Missing Data |
| Outcomes and Estimation | 17 | • Summary of results for each outcome, including effect sizes and confidence intervals | √ | Table 4, 5 |
|  |  | • Inclusion of null and negative findings | √ | Table 4, 6 |
|  |  | • Inclusion of results from testing pre-specified causal pathways through which the intervention was intended to operate, if any | x | NA |
| Ancillary Analyses | 18 | • Results of other analyses performed, including subgroup or restricted analyses • Indication of which were pre-specified, and which were exploratory | √ | Results |
| Adverse Events | 19 | • Summary of all important adverse events or unintended effects in each study condition (including harms, unintended benefits, and failures of intervention delivery) | √ | Results |
| Interpretation | 20 | • Interpretation of results, taking into account study hypotheses, sources of potential bias, imprecision, and multiplicity of analyses | √ | Discussion → Principal findings |
|  |  | • Discussion of results taking into account the mechanism by which the intervention was intended to work (causal pathways) or alternative mechanisms or explanations | √ | Discussion → Comparison to prior work |
|  |  | • Discussion of the success of and barriers to implementing the intervention, fidelity of implementation | √ | Discussion → Comparison to prior work + limitation and recommendations |
|  |  | • Discussion of research, programming, or policy implications | √ | Discussion → Public Health Policy Recommendations |
| Generalizability | 21 | • Generalizability (external validity) of trial findings | √ | Discussion → limitation and recommendations |
| Overall Evidence | 22 | • General interpretation of results in the context of current evidence and theory | √ | Conclusions |
